# The Administration of the New Pyrimidine Derivative—4-{2-[2-(3,4-Dimethoxyphenyl)-Vinyl]-6-Ethyl-4-Oxo-5-Phenyl-4H-Pyrimidine-1-Il}Benzsulfamide Restores the Activity of Brain Cells in Experimental Chronic Traumatic Encephalopathy by Maintaining Mitochondrial Function

**DOI:** 10.3390/medicina55070386

**Published:** 2019-07-17

**Authors:** Dmitry I. Pozdnyakov, Kirill A. Miroshnichenko, Andey V. Voronkov, Tat’yana G. Kovaleva

**Affiliations:** Pyatigorsk Medical and Pharmaceutical Institute, Volgograd State Medical University, Pyatigorsk 357532, Russia

**Keywords:** chronic traumatic encephalopathy, pyrimidine derivatives, mitochondrial function

## Abstract

*Background and objectives:* To evaluate the effect of a new pyrimidine derivative on the change of mitochondrial function in experimental chronic traumatic encephalopathy. *Materials and methods:* The study was performed on male mice of the BALB/c line (acute toxicity was assessed) and male rats of the Wistar line, which were modeled chronic traumatic encephalopathy by the method of free fall of the load (weight 150 g from a 50 cm height). The injury to rats was reproduced once a day for 7 days. Further, cognitive functions, changes in sensorimotor deficiency, cerebral blood flow, neuron-specific enolase(NSE), S100β, glial fibrillary acidic protein (GFAP) (in blood serum) and β-amyloid, adenosine triphosphate (ATP) (in brain tissue supernatant) were evaluated. Mitochondrial respiration was also measured. Choline alfoscerate (100 mg/kg) was used as a reference drug. *Results:* The study found that the use of a new pyrimidine derivative contributed to the preservation of the mitochondrial respirometric function and cognitive functions in rats. In addition, against the administration of test-object marked increase in the concentration of ATP, the velocity of cerebral blood flow was 4.2 times (*p* < 0.05) and 35.6% (*p* < 0.05), respectively, as well as reduced concentration and GFAP, NSE, S100β, β-amyloid and sensorimotor deficit at 2.7 (*p* < 0.05) times; 2 times (*p* < 0.05); 2.4 times (*p* < 0.05); of 30.4% (*p* < 0.05 and 46.5% (*p* < 0.05), respectively. The LD_50_ (per os) for the test-object was 4973.56 ± 573.72 mg/kg. *Conclusion:* Based on the obtained data, high therapeutic efficacy and low systemic toxicity of the application are assumed 4-{2-[2-(3,4-dimethoxyphenyl)-vinyl]-6-ethyl-4-oxo-5-phenyl-4H-pyrimidine-1-Il}benzsulfamide in chronic traumatic encephalopathy.

## 1. Introduction

Chronic traumatic encephalopathy (CTE) is a progressive neurodegenerative disease that occurs as a result of repetitive episodes of mild or moderately severe traumatic brain injury, the clinical manifestations of which are cognitive, sensorimotor and neurological deficits. The first reports on the development of CTE are dated to the first half of the 20th century when this pathology was diagnosed in professional boxers and was called “dementia pugilistica” [1]. Later, since 1940, the concept of CTE has been substantially supplemented, while CTE was found not only in people suffering from sports injuries of the brain but also in individuals susceptible to domestic injuries [2]. To date, it has been established that contact sports athletes (American football, rugby, hockey, boxing, mixed martial arts) and military personnel who have undergone traumatic brain injury as a result of the action of a blast wave or repetitive brain concussion constitute the “risk group” of CTE [3]. It is noted that these individuals have an increased risk of developing post-traumatic dementia, Parkinson’s and Alzheimer’s disease. The pathogenetic basis of CTE is the destruction of neurons as a result of repeated exposure to traumatic factors and the accumulation of β-amyloid in the brain tissue [4]. In this case, as noted by McKee et al., 2013, the deposition of β-amyloid plaques in the brain leads to generalized vasculopathy, resulting in increased ischemic-hypoxic processes in the brain tissues [5]. In turn, a decrease in the level of cerebral blood flow, as well as an intensification of hypoxic phenomena, leads to a worsening of the aerobic metabolism reactions in brain tissue, which aggravates the course of the pathological process [6]. At the same time, in conditions of insufficient oxygenation of the brain tissue, the mitochondria of neurons are primarily damaged, which in turn increases the manifestation of oxidative stress, energy deficiency and intensifies the cascade of apoptosis reactions [7]. In view of the lack of effective medicines for pharmacological correction of CTE, mitochondrial targeting may be a promising basis for the creation of new effective and safe drugs for the CTE treatment.

## 2. Materials and Methods

### 2.1. Experimental Animals

The study was performed on 70 BALB/c male mice weighing 20 ± 2 g and 40 Wistar male rats weighing 240–260 g. At the time of the experiment, the animals were kept under controlled conditions at an ambient temperature of 22 ± 2 °C, a relative humidity of 60% ± 5% and an inartificial change of the daily cycle. The animals were kept in macrolon boxes by five individuals, which excluded crowding and stress. Granulated beech fraction was used as bedding material. Access to water and food for animals was not restricted. The contents and all manipulations with animals complied the requirements of the European Convention for the Protection of Vertebrate Animals used for experiments and other scientific purposes (Strasbourg, 1986). The study was approved by the local ethics committee (protocol No. 15 from 20 March 2019).

### 2.2. Test Compound and Reference Drugs

**Figure 1:**
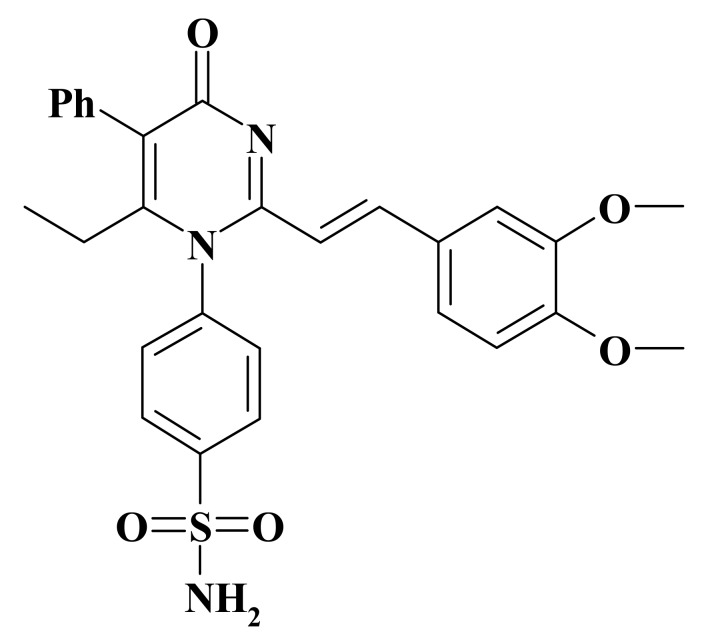
Structure of the test compound.

The studied compound was a new pyrimidine derivative: 4-{2-[2-(3,4-dimethoxyphenyl)-vinyl]-6-ethyl-4-oxo-5-phenyl-4H-pyrimidine-1-Il}benzsulfamide, laboratory code OCH (Figure 1). The structure of the test-object was confirmed by NMR spectroscopy. In this study, choline alfoscerate at a dose of 100 mg/kg was used as a reference drug for a group of rats (*n* = 10) as described previously [8]. When simulating CTE, the test compound was administered at the dose of 100 mg/kg (group of rats *n* = 10) at an equivalent dose to reference drug. The test object and the reference drug were administered orally (the animal was fixed by hand and the test-compound and the reference drug were administered in the indicated doses through the atraumatic needle with tin extension to the end) for 7 days once in a day after 30 min from injury. In addition, during the study, an intact (*n* = 10) and a group of negative control rats (NC, *n* = 10) were formed.

### 2.3. Determination of Single-Dose Toxicity of the Test-Substance

At the first stage, the single-dose toxicity of the test compound was evaluated, where BALB/c male mice were used as a biological model. In determining single-dose toxicity, the test compound was administered per os in the dose range: 250 mg/kg; 500 mg/kg; 1000 mg/kg; 1500 mg/kg; 2000 mg/kg and 2500 mg/kg. At the same time, the level of mortality and the general condition of animals were recorded for 14 days. Based on the obtained data, the LD_50_ index was calculated according to the Finney method [9].

### 2.4. Model of Chronic Traumatic Encephalopathy

Chronic traumatic encephalopathy (CTE) was reproduced in male rats of the Wistar line by the method of a free fall of a 150 g load from a height of 50 cm to the parietal region of the rat skull. The injury was reproduced once a day for 7 days [10,11]. Functional tests and biomaterial sampling were performed on the 8th day of the experiment.

### 2.5. Test “Conditioned Reflex of Passive Avoidance—CRPA”

The device consisted of two adjacent compartments, illuminated with dimensions of 60 × 40 cm and darkened with dimensions of 15 × 15 cm, equipped with an electrostimulating floor and connected to a large chamber with a square hole of 16 cm^2^ area. Testing procedure: the rat was placed in the middle of the site of the illuminated chamber, its tail to the dark compartment, and for 2 min the latent period of the first entry into the dark compartment was recorded, where the animal received electro-balance irritation (three pulses by 40 V). Before reproducing CTE, animals were trained in the testing procedure.

### 2.6. Test “Extrapolation Avoidance—TEA”

The experimental device was a polymer cylinder with a diameter of 35 cm filled with water (20 °C). In the center of the external cylinder an internal acrylic cylinder with a diameter of 9 cm and a height of 22 cm was placed, the lower edge of which is immersed in water to a depth of 2.5 cm. Testing procedure: the animal was placed in the internal cylinder and its behavior was recorded (the number of unsuccessful attempts to avoid) and the latent time of the task (“diving”; “avoidance”) for 2 min. Before reproducing CTE, animals were trained in the testing procedure.

### 2.7. Determination of Sensorimotor Deficiency

The sensorimotor deficit was evaluated at the Beam Walking test. The device consisted of a tapering path, 165 cm long, with sides for fixing the fall of animal limbs and a dark chamber at the end of the path. The starting point of the animals was illuminated by a bright light, motivating the animal to move toward the ultimate goal—the dark chamber. Previously (before playing CTE), animals were taught the test procedure for 4 days. After the CTE simulation, animals were re-tested to determine the degree of sensorimotor deficiency, and the number of complete limb setting aboard and the number of incomplete limb setting on board (“errors”) were recorded. Sensomotor deficit was calculated using Equation (1) [12].

Sensomotor deficiency, % = (number of complete limb setting on board × 1 + number of “errors” × 0.5) ÷ total number of steps × 100%(1)

### 2.8. Determination of Cerebral Blood Flow Rate

The cerebral blood flow rate in rats was assessed using the Doppler ultrasound method. In this work, a USOP-010-01 sensor with an operating frequency of 25 MHz and the MM-D-K-Minimax Doppler v.2.0 software package were used. (Saint-Petersburg, Russia). The analysis determined the change in the average systolic linear velocity of cerebral blood flow in the parietal region of the brain of rats. For this purpose, in the parietal area of the animals cranium, a trepanning hole 3 mm in diameter was made and a Doppler sensor was installed. An acoustic gel was used as a contact sound-conducting medium [13].

### 2.9. Biomaterial Sample Preparation

The brain and blood serum of experimental animals were used as a biomaterial. In anesthetized rats (chloralgitrate, 350 mg/kg, intraperitoneally), blood was drawn from the abdominal aorta into a syringe with a citrate filling. The collected blood was centrifuged at 1000× *g* for 15 min, the serum was withdrawn and used for enzyme-linked immusorbent assay (ELISA). Subsequently, the animals were decapitated, the skull was opened, and the brain was removed, which was homogenized in cold using a Potter mechanical homogenizer in 0.01 mmol/L PBS with a pH of 7.2 (1:7 ratio). The resulting homogenate was centrifuged at 10,000× *g* for 5 min. Brain tissue supernatant was removed for ELISA. For respiratory analysis the brain was collected, after which the biomaterial was homogenized in a Potter mechanical homogenizer in a selection medium (1 mmol EDTA, 215 mmol mannitol, 75 mmol sucrose, 0.1% BSA solution, 20 mmol HEPES, with a pH of 7.2). The cell population was obtained by differential centrifugation, for which the obtained biogenic homogenate was centrifuged at 1400× *g* for 3 min, at 4 °C, after which the supernatant was transferred to 2 mL tubes. Next, the resulting supernatant was centrifuged at 13,000× *g* for 10 min and the supernatant (culture contains native mitochondria) was removed for analysis [14].

### 2.10. Respirometric Analysis

Analysis of the state of the respiratory function of mitochondria was carried out by the method of respirometry using the AKPM1-01L laboratory respirometer system (Alfa Bassens, Russia). The mitochondrial respiratory function was assessed by the change in oxygen consumption in the medium against the introduction of mitochondrial respiratory uncouplers: oligomycin 1 µg/mL; 4-(trifluoromethoxy) phenyl) hydrazono) malononitrile (FCCP-1 µM); rotenone—1 µM; sodium azide—20 mmol. The overall assessment of mitochondrial function was determined by the level of oxygen consumption in the medium after sequential addition of oligomycin, FCCP and rotenone to the medium, and the ATP-generating ability was determined (by the difference in oxygen consumption after the addition of FCCP and oligomycin); the maximum level of respiration (according to the difference in oxygen consumption after the addition of FCCP and rotenone) and the respiratory capacity (according to the difference in oxygen consumption after the addition of FCCP and the basal level of oxygen consumption). The activity of the glycolysis processes was evaluated when glucose was used as an oxidation substrate during the registration of oxygen consumption under the conditions of sequential addition of glucose, oligomycin and sodium azide to the medium. The intensity of glycolysis was determined (according to the difference in oxygen consumption after adding glucose and the basal level of oxygen consumption), glycolytic capacity (according to the difference in oxygen consumption after adding oligomycin and glucose) and glycolytic reserve (according to the difference in oxygen consumption after adding glucose and sodium azide). During the analysis, the biosample volume was 275 μL, and 25 μL of injected analyzers. Oxygen consumption was determined in ppm [15].

### 2.11. ELISA Study

The concentration of phosphorylated β-amyloid and ATP in the supernatant was determined by ELISA. In the blood serum, changes in the content of S100β protein, glial fibrillary acidic protein (GFAP) and neuron-specific enolase (NSE) were evaluated. The analysis was performed using standard Cloud clone (Cloud-Clone Corp., Katy, TX, USA) reagent kits. The course of the analysis corresponded to the instructions attached to each kit.

### 2.12. Statistical Analysis

Statistical processing of the obtained results was performed using the STATISTICA 6.0 software package. (StatSoft, Tulsa, OK, USA) Data are expressed as M ± SEM. Comparison of means was carried out by the one-way analysis of variance (ANOVA) method with the post-test of Newman-Keuls. Differences were considered statistically significant at *p* < 0.05.

## 3. Results

### 3.1. Evaluation of the Single-Dose Toxicity of the Test-Compound

The results of the study of the single-dose toxicity of the test-object under the code of OCH are presented in Table 1. During this block of experimental work, it was found that there administration test-compound in doses of 250–1500 mg/kg did not cause animal death. At the same time, when OCH was administered in doses of 2000 mg/kg and 2500 mg/kg, one animal out of 10 died (with OCH administered in a dose of 2000 mg/kg on the 4th day of the study, and 2500 mg/kg was administered on the 6th day of the experiment). At the same time, the surviving animals throughout the observation period (14 days) of sensorimotor, behavioral and functional abnormalities relative to the control group (*n* = 10) were not established. Thus, the LD_50_ for the OCH compound was 4973.56 ± 573.72 mg/kg, which made it possible to assign this compound to the 5th hazard class according to the GHS classification.

### 3.2. The Effect of the OCH Compound on the Development of Cognitive and Sensorimotor Deficiency in Rats under CTE Conditions

The study found that in the negative control group under conditions of CTE there was a violation of cognitive-mnestic functions, as evidenced by the obtained data in the CRPA and TEA tests. In the CRPA test in the NC group compared to the intact group, a decrease in the latent time of entry into the dark compartment of the device by 10.4 times (*p* < 0.05) was obtained (Table 2).

In rats lacking pharmacological support for intact animals in the TEA test (Table 3), there was an increase in the time of “avoidance” by 5.2 times (*p* < 0.05) and the number of unsuccessful attempts to complete the task by 4.7 times (*p* < 0.05).

**Figure 2:**
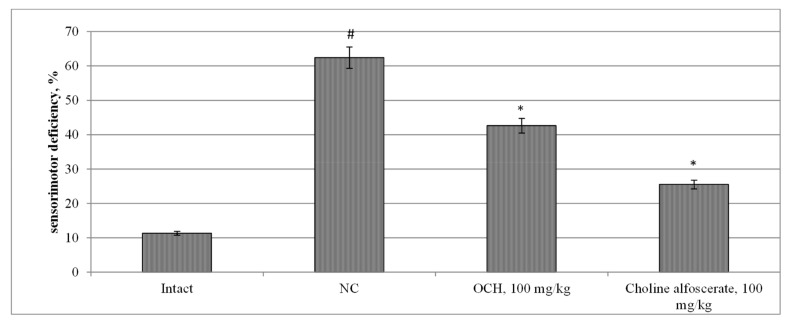
The effect of the OCH compound and choline alfoscerate on the change of sensorimotor deficiency in rats under experimental CTE conditions. Note: *—statistically significant relative to the NC group of animals; #—statistically significant relative to the intact group of animals.

It should be noted that cognitive impairment in rats under CTE conditions was accompanied by the development of sensorimotor impairments, as evidenced by an increase in sensomotor deficit (Figure 2) in NC group relative to intact rats by 5.5 times (*p* < 0.05). Against the background of the use of choline alfoscerate, an increase (by 5.7 times (*p* < 0.05)) in the latent time of animals entering the dark compartment of the device in the test CRPA was noted, as well as a decrease in the time to make a decision in test TEA (“avoidance”) by 113, 3% (*p* < 0.05), while the number of unsuccessful “avoidance” attempts in rats treated with choline alfostserat did not change statistically significantly with respect to the NC group (Table 3).

The use of choline alfosecerate in experimental CTE contributed to a 2.4 reduction in sensorimotor deficiency in comparison with an NC group (*p* < 0.05) (Figure 2). The use of the OCH compound in CTE conditions contributed to a reduction in the time of avoidance and the number of unsuccessful attempts to perform the task in rats in the TEA test relative to the NC group by 58.3% (*p* < 0.05) and 2.6 times (*p* < 0.05) respectively. In the CRPA test, in rats treated with the compound OCH, with respect to animals deprived of pharmacological support, an increase in the latent time of entry into the dark compartment of the device by 5.7 times (*p* < 0.05) was noted (Table 1). In addition, when the compound OCH was administered to rats, the sensorimotor deficit was 46.5% (*p* < 0.05) less than that of the NC group (Figure 2).

### 3.3. The Effect of the OCH Compound on the Change in the Rate of Cerebral Blood Flow in Rats under Conditions of CTE

**Figure 3:**
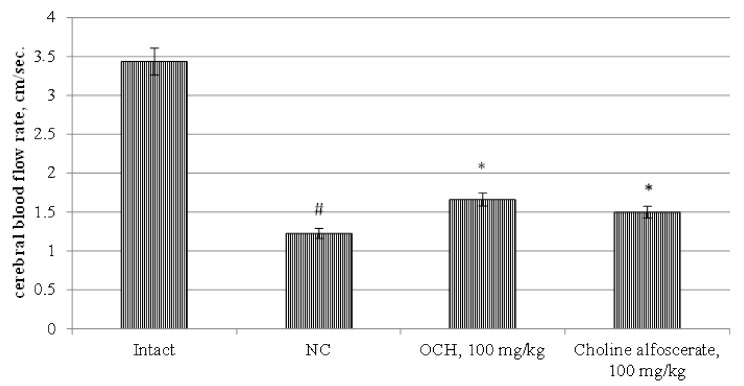
The effect of the OCH compound and choline alfoscerate on the change of cerebral blood flow rate in rats under experimental CTE conditions. Note: *—statistically significant relative to the NC group of animals; #—statistically significant relative to the intact group of animals.

The intact group had a cerebral blood flow rate of 3.344 ± 0.234 cm/s. (Figure 3). At the same time, in the NC group, in comparison with intact rats, there was a decrease in the level of cerebral blood flow by 2.8 times (*p* < 0.05). The introduction of choline alfoscerate and the OCH compound into animals showed an increase in cerebral blood flow rate in comparison with the NC group by 22.3% (*p* < 0.05) and 35.6% (*p* < 0.05), respectively (Figure 3).

### 3.4. The Effect of the OCH Compound on the Change in the Concentration of Specific Markers of CTE in Rats under Conditions of Experimental Pathology

During this block of the study, it was found that experimentally reproduced CTE in rats was accompanied by an increase in the concentration of GFAP, NSE and S100β (Table 4) in the blood serum of animals relative to intact rats by 8.4 times (*p* < 0.05); 18 (*p* < 0.05) and 24.3 (*p* < 0.05) times, respectively, with an increase in the β-amyloid content in the brain supernatant of the NC group by 30.6 times (*p* < 0.05). In rats treated with choline alfoscerate, relative to the group of negative control animals, a decrease in serum GFAP, NSE and S100β levels was observed by 3.1 times (*p* < 0.05); 2.4 times (*p* < 0.05) and 2.4 times (*p* < 0.05), respectively (Table 4). At the same time, the content of β-amyloid in brain tissue in rats that were administered choline alfoscerate was 4.1 times (*p* < 0.05) less than the analogous parameter of animals of the NC group. A decrease in the blood serum GFAP, NSE and S100β concentrations on animals treated by OCH compound compared with the NC group by the 2.7 (*p* < 0.05) times; 2 times (*p* < 0.05) and 2.4 times (*p* < 0.05), respectively was noted. In addition, the use of the OCH compound contributed to a decrease in the β-amyloid content (Table 4) in the supernatant of the rat brain tissue by 30.4% (*p* < 0.05).

### 3.5. The Effect of OCH Compound on the Change of Mitochondrial Function in Rats under CTE Conditions

**Figure 4:**
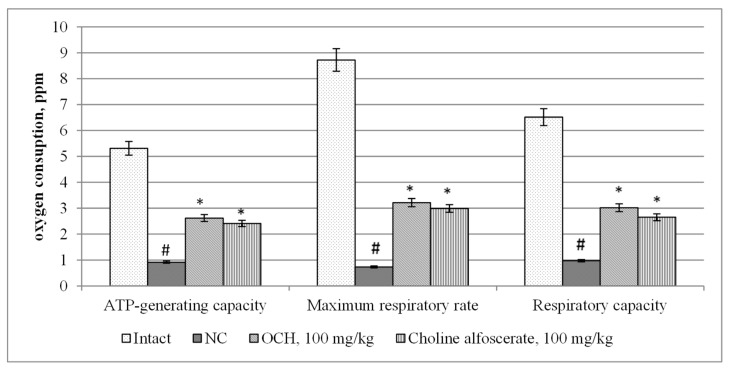
The effect of the OCH compound and choline alfoscerate on the change of the mitochondrial respiration in rats under conditions of experimental CTE. Note: *—statistically significant relative to the NC group of animals; #—statistically significant relative to the intact group of animals.

**Figure 5:**
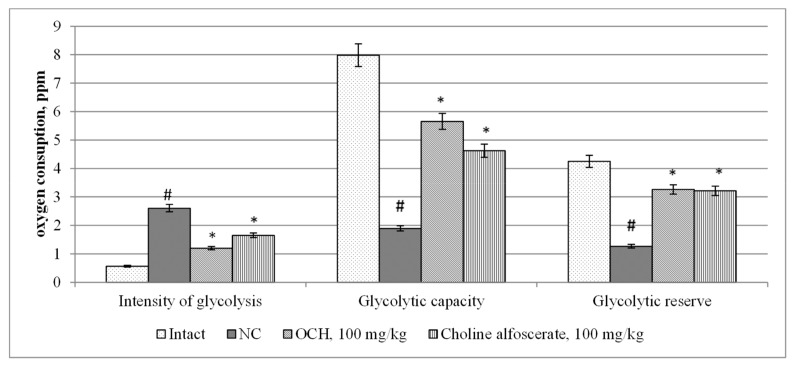
The effect of the OCH compound and choline alfoscerate on the change in the intensity of glycolysis processes in rats under experimental CTE conditions. Note: *—statistically significant relative to the NC group of animals; #—statistically significant relative to the intact group of animals.

**Figure 6:**
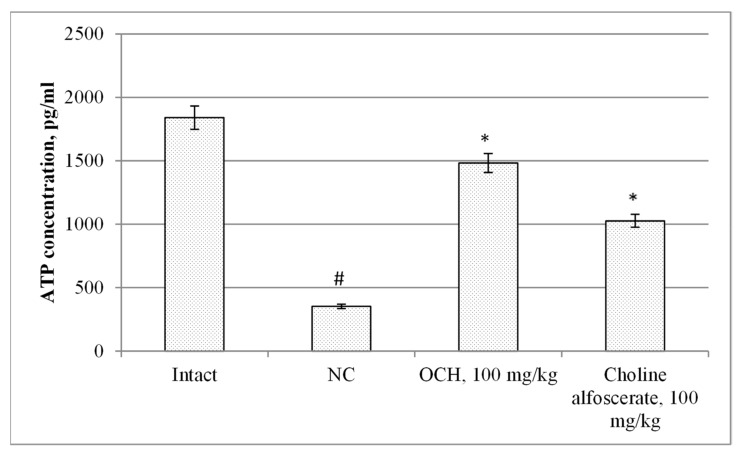
The effect of the OCH compound and choline alfoscerate on the change in ATP concentration in the brain supernatant in rats under experimental CTE condition. Note: *—statistically significant relative to the NC group of animals; #—statistically significant relative to the intact group of animals.

When assessing changes in mitochondrial function in animals under experimental CTE conditions, it was found that the NC group compared to intact rats, there was a decrease in the ATP-generating activity of mitochondria, the maximum respiratory rate and respiratory capacity (Figure 4) by 5.8 (*p* < 0.05); 11.9 (*p* < 0.05) and 6.7 (*p* < 0.05) times, respectively. At the same time, in the NC group with respect to intact animals, an increase in glycolysis intensity by 4.6 times (*p* < 0.05), and a decrease in glycolytic capacity and glycolytic reserve by 4.2 times (*p* < 0.05) and 3.3 times (*p* < 0.05), respectively (Figure 5) was noted. As a result, the ATP content (Figure 6) in the brain supernatant of the NC group decreased by 5.2 (*p* < 0.05).

Against the background of the use of choline alfoscerate in animals compared with the NC group, an increase in the ATP-generating ability, maximum respiration rate and respiratory capacity (Figure 4) by 2.6 (*p* < 0.05); 4.1 times (*p* < 0.05) and 2.7 times (*p* < 0.05), respectively, while reducing the intensity of glycolysis 1.6 times (*p* < 0.05) and increasing the glycolytic capacity and glycolytic reserve by 2.4 times (*p* < 0.05) and 2.5 times (*p* < 0.05), respectively (Figure 5) was observed. The ATP content in the brain tissue of animals treated by choline alfoscerate increased by 2.9 times (*p* < 0.05) compared with the analogous index of NC groups (Figure 6).

When the test-compound was administered to animals relative to an NC group, an increase in ATP-generating activity, maximum respiration rate (Figure 4), respiratory capacity by 2.9 times (*p* < 0.05); 4.4 (*p* < 0.05) and 3.1 times (*p* < 0.05), respectively was observed. In addition, in animals receiving the OCH compound, the intensity of glycolysis (Figure 5) was 2.2 times (*p* < 0.05) lower than that in the NC group, and the glycolytic capacity and glycolytic reserve were, respectively, higher by 3 (*p* < 0.05) and 2.6 (*p* < 0.05) times. As a result, the ATP concentration (Figure 6) in the supernatant of the brain tissue of animals that were treated by OCH compound surpassed that of the NC group by 4.2 times (*p* < 0.05).

## 4. Discussion

The risk of developing CTE among military personnel is also high [16]. The molecular basis of the pathogenesis of CTE is a combination of the processes observed as a result of primary (irreversible damage) and secondary (reversible) damage of brain tissue. Irreversible destructive processes in the brain tissue, resulting from the action of the traumatic factor, initiate secondary damage to the brain tissue, including: edema, increased intracranial pressure and hemorrhage, decreased cerebral blood flow, metabolic disorders, glutamate excitotoxicity, calcium overload, oxidative stress, mitochondrial dysfunction, inflammation and activation of the proapoptotic signal [17]. At this stage of the disease, clinical manifestations are absent, and the pathophysiological cascade of reactions closes on the formation of neurotoxic β-amyloid and neurofibrillary tangles of phosphorylated tau-protein, which is one of the main negative prognostic factors of CTE [18]. Due to the impossibility of clinically diagnosing CTE at early stages, the methods of treatment of this pathological condition are practically absent or are under development. Most of the currently described potential pharmacotherapeutic approaches to the treatment of CTE relate to the targeted correction of tau-pathology. Therefore, in a study conducted by Kondo et.al. 2015, it was shown that the use of specific antibodies to tau-protein reduced progressive tau-pathology after a traumatic brain injury [19]. In addition, the work carried out by Zhang J, et.al., 2015, made it possible to establish that inhibition of monoacylglycerol lipase prevented the formation of the phosphorylated tau-protein form in the brain of mice with reproducible CTE [20]. As can be seen, at present, the number of effective methods for treating CTE is fairly limited, which may be a prerequisite for the development of new pharmacological targets and drugs for treating CTE, the use of which can significantly improve the epidemiological situation with chronic brain injury. This promising pharmacological target may be neuronal mitochondria [21]. Mitochondria play a crucial role in bioenergy, redox state and cell survival [22]. As the neuronal damage progresses due to the action of the traumatic factor, i.e., a primary cascade of destructive reactions, the involvement of mitochondria in the pathological process is becoming increasingly important. It has been established that dysfunction of mitochondria resulting from tissue ischemia leads to a progressive decrease in ATP synthesis, activation of free-radical oxidation reactions [23]. In addition to energy deficiency and oxidative stress, damage to mitochondria leads to an increase in the intensity of caspase-dependent (effector-caspase-3) and caspase-independent (effector-endonuclease G) apoptosis reactions—the main mechanisms of cellular destruction during the secondary brain damage as a result of injury [24]. Thus, the preservation of optimal mitochondrial function can contribute to the reduction of cellular destruction in the conditions of CTE at the earlier stages of the pathogenetic cascade of brain damage.

Based on the analysis of the literature, the concept of this study was formulated, suggesting the hypothesis that the use of a pyrimidine derivative: 4-{2-[2-(3,4-dimethoxyphenyl)-vinyl]-6-ethyl-4-oxo-5-phenyl-4H-pyrimidin-1-yl}benzylsulfamide under conditions of experimental CTE, will soften the course of the disease by restoring mitochondrial function and normalizing ATP synthesis. The basis for the selection of the test-object was the previously established cerebroprotective properties of pyrimidine derivatives under conditions of acute traumatic brain injury [25].

The study found that the use of 4-{2-[2-(3,4-dimethoxyphenyl)-vinyl]-6-ethyl-4-oxo-5-phenyl-4H-pyrimidin-1-yl}benzosulfamide (laboratory code OCH) contributed to an increase in ATP-generating activity, the maximum level of respiration, respiratory capacity by 2.9 times (*p* < 0.05); 4.4 (*p* < 0.05) and 3.1 times (*p* < 0.05), respectively, as well as a decrease in the glycolysis intensity (2.2 times (*p* < 0.05)), an increase in glycolytic capacity and glycolytic reserve in 3 (*p* < 0.05) and 2.6 (*p* < 0.05) times, respectively. As a result, the ATP concentration in the supernatant of the brain tissue of animals that were treated by OCH was by 4.2 times (*p* < 0.05) higher than that of a group of rats deprived of pharmacological support. It should be noted that the indicators of mitochondrial function in animals treated with the studied compound, and the reference drug choline alphoscerate did not differ significantly from each other. It was also found that the use of the OCH compound under CTE conditions promoted a decrease in the concentration of GFAP, NSE, S100β, and β-amyloid in comparison with animals of the NC group by 2.7 (*p* < 0.05) times; 2 times (*p* < 0.05); 2.4 times (*p* < 0.05) and by 30.4% (*p* < 0.05). respectively (GFAP, NSE, S100β—determined in blood serum; β-amyloid—in the supernatant of brain tissue). It is known that GFAP, NSE and S100β are important biomarkers, allowing with sufficient probability to determine the severity of CTE and its outcome [26]. At the same time, an increase in serum GFAP is a sign of diffuse brain damage and indicates damage to glial cells [27], and an increase in the concentration of NSE suggests a progressive nature of neurodegeneration [28].

Thus, a decrease in the concentration of GFAP, NSE and S100β against the background of the use of an OCH compound may indicate a decrease in diffuse brain damage and preservation of the integrity of glial cells. At the same time, a decrease in the β-amyloid content when using the tested compound can be a sign of a reduction in the progression of tau-pathology and the accumulation of phosphorylated tau-protein [29]. In addition, on the background of the administration of the studied compound into animals, the recovery of cerebral blood flow in rats was noted (increased by 35.6% (*p* < 0.05)). Observed biochemical and functional changes in the brain may underlie the improvement in clinical performance in animals treated with OCH under CTE conditions. The use of this compound contributed to the preservation of the memorable trace in rats, which was confirmed by the results of the CRPA and TEA tests. In addition, the sensorimotor deficiency rate in animals on the background of the administration of the OCH compound decreased by 46.5% (*p* < 0.05) in relation to rats deprived of pharmacological support. At the same time, it is of considerable importance that the test substance OCH is a low-toxic compound (the LD_50_ parameter was 4973.56 ± 573.72 mg/kg at oral administration).

## 5. Conclusions

Based on the obtained data, it can be assumed that the pyrimidine derivative: 4-{2-[2-(3,4-dimethoxyphenyl)-vinyl]-6-ethyl-4-oxo-5-phenyl-4H-pyrimidin-1-yl}benzosulfamide contributes to the restoration of the functional and metabolic activity of brain cells in rats under conditions of experimental CTE. In this case, the mechanism of action of 4-{2-[2-(3,4-dimethoxyphenyl)-vinyl]-6-ethyl-4-oxo-5-phenyl-4H-pyrimidin-1-yl}benzosulfamide may be based on the preservation of mitochondrial functions. The significant level of efficacy associated with low toxicity of the application makes the tested pyrimidine derivative a promising means of pharmacological correcting of CTE.

## Figures and Tables

**Table 1:** The results of the evaluation of the single-dose toxicity of the 4-{2-[2-(3,4-dimethoxyphenyl)-vinyl]-6-ethyl-4-oxo-5-phenyl-4H-pyrimidine-1-Il}benzsulfamide (OCH) compound.

Dose, mg/kg	The Number of Animals in the Group	% Mortality	LD_50_, mg/kg
250	10	0	4973.56 ± 573.72
500	10	0	
1000	10	0	
1500	10	0	
2000	10	10	
2500	10	10	
Control	10	-	-

**Table 2:** The effect of the OCH compound and choline alfoscerate on the behavioral activity of rats in the conditioned reflex of passive avoidance test.

Group	The Latent Time of Entry into the Dark Compartment
Intact	110.5 ± 2.754
NC	10.67 ± 1.645 ^#^
OCH, 100 mg/kg	58.2 ± 2.192 *
Choline alfoscerate, 100 mg/kg	30.21 ± 2.531 *

* statistically significant relative to the NC group of animals; ^#^ statistically significant relative to the intact group of animals.

**Table 3:** The effect of the OCH compound and choline alfoscerate on the behavioral activity of rats in the extrapolation avoidance test.

Group	Avoidance Time	The Number of Unsuccessful Attempts to Avoid
Intact	10.23 ± 1.264	4.6 ± 0.561
NC	52.25 ± 2.343 ^#^	21.5 ± 7.773 ^#^
OCH, 100 mg/kg	33 ± 0.949 *	8.2 ± 1.828 *
Choline alfoscerate, 100 mg/kg	24.5 ± 1.414 *	23 ± 1.414 *

Note: *—statistically significant relative to the NC group of animals; ^#^—statistically significant relative to the intact group of animals.

**Table 4:** The effect of the OCH compound and choline alfoscerate on the change in the concentration of specific biomarkers of neuronal destruction under the conditions of experimental CTE.

Group	GFAP, pg/mL	β-amyloid, pg/mL	S100β, pg/mL	NSE, pg/mL
Intact	299.3 ± 9.265	10.21 ± 0.691	13.23 ± 0.229	332.12 ± 5.531
NC	2526.2 ± 52.321 ^#^	312.31 ± 1.945 ^#^	321.01 ± 10.016 ^#^	6124 ± 35.156 ^#^
OCH, 100 mg/kg	925.7 ± 26.951 *	239.45 ± 10.322 *	132.1 ± 12.484 *	2504.32 ± 21.362 *
Choline alfoscerate, 100 mg/kg	806.5 ± 31.011 *	76.91 ± 13.264 *	128.34 ± 13.065 *	3021 ± 28.314 *

Note: *—statistically significant relative to the NC group of animals; ^#^—statistically significant relative to the intact group of animals.
